# Solving the Multi-Functional Heterogeneous UAV Cooperative Mission Planning Problem Using Multi-Swarm Fruit Fly Optimization Algorithm

**DOI:** 10.3390/s20185026

**Published:** 2020-09-04

**Authors:** Rubin Luo, Hongxing Zheng, Jifeng Guo

**Affiliations:** 1 Institute of Aerospace Systems Engineering, Beijing 100076, China; armybin@163.com; 2 School of Astronautics, Harbin Institute of Technology, Harbin 150001, China; zhenghongxing_hit@aliyun.com

**Keywords:** multi-functional heterogeneous UAVs, cooperative mission planning, multi-swarm fruit fly optimization algorithm

## Abstract

The complexity of unmanned aerial vehicle (UAV) missions is increasing with the rapid development of UAV technology. Multiple UAVs usually cooperate in the form of teams to improve the efficiency of mission execution. The UAVs are equipped with multiple sensors with complementary functions to adapt to the complex mission constraints. Reasonable task assignment, task scheduling, and UAV trajectory planning are the prerequisites for efficient cooperation of multi-functional heterogeneous UAVs. In this paper, a multi-swarm fruit fly optimization algorithm (MFOA) with dual strategy switching is proposed to solve the multi-functional heterogeneous UAV cooperative mission planning problem with the criterion of simultaneously minimizing the makespan and the total mission time. First, the multi-swarm mechanism is introduced to enhance the global search capability of the fruit fly optimization algorithm. Second, in the smell-based search phase, the local search strategies and large-scale search strategies are designed to drive multiple fruit fly swarms, and the dual strategy switching method is presented. Third, in the vision-based search stage, the greedy selection strategy is adopted. Finally, numerical simulation experiments are designed. The simulation results show that the MFOA algorithm is more effective and stable for solving the multi-functional heterogeneous UAV cooperative mission planning problem compared with other algorithms.

## 1. Introduction

The application of unmanned aerial vehicles (UAV) in military and civil fields is growing rapidly, such as for pesticide spraying, forest fire prevention, material delivery, reconnaissance, etc. Low cost and high safety are the two main driving forces for the rapid development of unmanned aerial vehicles. The unmanned aerial vehicle control method is very flexible [1,2], specifically including the following: (i) human operator remote control; (ii) pre-programmed or uploaded mission plan via communication device; (iii) UAV autonomous operation. The improvement of the autonomous level of unmanned aerial vehicles can effectively reduce the operator’s burden and the probability of mistakes, while improving the efficiency and quality of mission completion. Moreover, for complex tasks such as large-scale information gathering, regional reconnaissance, and search and rescue, the cooperation of multiple UAVs in the form of a team is usually required. The multi-UAV cooperative mission planning problem (MCMPP) plays an essential role in the autonomous operation of multiple UAVs, which is a non-deterministic polynomial time (NP)-hard combinatorial optimization problem. The MCMPP involves rationally assigning the UAVs and scheduling the tasks on the premise of meeting the constraints of the task sequence, spatial location, energy supply, etc. to achieve the optimization of the established mission planning objectives.

Task assignment is an essential sub-problem of the MCMPP, which was extensively studied in robotics, UAVs, operations research, etc. From the modeling perspective, the task assignment model is very relevant to classic combinatorial optimization problems, such as vehicle routing problem (VRP) [3], job-shop scheduling problem (JSP) [4], traveling salesman problem [5], team orienteering problem [6], etc. Classic combinatorial optimization problems or variant problems can be transformed into mathematical models for missions such as cooperative reconnaissance, data collection, search and rescue, etc. Faigl [7] formulated the data collection problem as a variant of the combinatorial optimization problems, i.e., the price-collecting traveling salesman problem (PCTSP) and the orienteering problem (OP). An unsupervised learning method called growing self-organizing array was proposed, which only takes tens of milliseconds to solve the information collection problem with a large number of sensor nodes. Yu et al. [8] proposed the correlated orienteering problem for modeling persistent monitoring tasks with time-invariant spatial correlations using autonomous mobile robots.

From the perspective of the solution method, exact and heuristic methods are practical solutions. Exact methods (e.g., branch-and-bound [9], branch-and-price [10,11], etc.) are optimal, but the computational complexity is proportional to the scale of the problem. Therefore, exact methods are only suitable for solving the small-sized problems. In order to reduce the difficulty of solving problems caused by the scale of the problem, heuristic methods (e.g., genetic algorithms (GA) [12,13,14,15], particle swarm optimization [16], differential evolution algorithm [17], etc.) have always been an essential solution to the MCMPP and combinatorial optimization problem. Ramirez-Atencia et al. [18] presented a new multi-objective genetic algorithm for solving complex mission planning problems involving a team of UAVs and a set of ground control stations. A new odor concentration calculation function was designed in conjunction with the constraint satisfiability problem, which accelerates the problem’s convergence rate. Chen et al. [19] presented a modified multi-objective symbiotic organism search algorithm for solving the reconnaissance task assignment problem for multiple UAVs with different sensor capacities. Chen et al. [20] presented a modified two-part wolf pack search algorithm to solve the multi-UAV cooperative task assignment problem.

Path planning is another critical sub-problem of the MCMPP. Due to its broad application in various fields (such as data collection, forest fire prevention, and military), the path planning problem was studied extensively. Path planning algorithms can be mainly divided into the following categories: graph-based search method [21], sampling-based method [22,23], and heuristics method [24]. The Dubins method is a classic graph-based search method, first introduced by Dubins [25] and later inspiring several studies. Chitsaz et al. [26] proposed the Dubins airplane model, an extension of the Dubins method, which aims to find the UAV’s optimal trajectory in three-dimensional space. Isaiah et al. [27] presented two motion planning algorithms for solving the Dubins travelling salesperson problem. Owen et al. [28] presented a system architecture for implementing the Dubins airplane model for a fixed-wing UAV. Macharet et al. [29] combined a metaheuristic algorithm with the Dubins method to solve the robot path planning problem.

Some research on the MCMPP problem integrated task assignment and path planning to design a unified system framework and generated a flyable path for the UAV while reasonably allocating team resources. Rasmussen et al. [30] proposed a tree search algorithm for solving the CMTAP, formulated in the form of a decision tree. Edison et al. [31] considered that multiple UAVs must perform reconnaissance, attack, and verify tasks on multiple mission targets. Task assignment and path planning are integrated and solved by genetic algorithm. Deng et al. [32] proposed a modified genetic algorithm with multiple gene types for solving the CMTAP. In the mission scenario, UAVs have different operational capabilities and kinematic constraints and carry limited resources. Weinan et al. [33] presented a coupled and distributed planning method based on the distributed genetic algorithm, using a fixed-wing UAV model with six degrees of freedom to verify the method.

In this paper, the multi-functional heterogeneous UAV cooperative mission planning problem (MFHCMPP) is proposed, as an extension of the MCMPP. In the MFHCMPP, each UAV has multiple functions with specific levels. Each task cannot be executed unless the UAV has the functions required by the task, and the functional level is not less than the standard functional level. In addition to the constraints of functions and functional levels, timing constraints exist between tasks, i.e., some tasks cannot be executed before their predecessors are completed. The UAV heterogeneity is mainly reflected in the difference in function, caused by different sensors or loads loaded by the UAVs. It is worth noting that the tasks are associated with some specific locations; thus, the task assignment of UAVs, the order of task execution, and the heading angle of the UAV approaching a task location will affect the length of the UAV flight path. The MFHCMPP problem proposed in this paper mainly solves three problems: task assignment, task scheduling, and path planning.

To solve the MFHCMPP problem, we propose the multi-swarm fruit fly optimization algorithm (MFOA). As an emerging swarm intelligence optimization algorithm, fruit fly optimization (FOA), in recent years, became one of the hottest research algorithms. The mechanism of FOA stems from a simulation of the process of searching for food sources in the fruit fly population. Based on the principles of olfactory search and visual search in the foraging process of fruit flies, the fruit fly optimization algorithm is mainly divided into two stages, namely, the smell-based search stage and the vision-based search stage. The two-stage continuous iterative optimization achieves the approach of the fruit fly population to the food source, before finally obtaining an optimal or satisfactory solution. Xiangyin et al. [34] proposed an improved fruit fly optimization (FOA) to solve the unmanned aerial vehicle (UAV) path planning problem, and the mutation adaptation mechanism was adopted to enhance the search ability of FOA and prevent it from falling into a local optimum. Xiaolong et al. [35] proposed a knowledge-based fruit fly optimization algorithm for solving the multi-skill resource-constrained project scheduling problem. A knowledge-based search procedure was introduced to enhance exploration. The knowledge of a fruit fly comes from the experience of the evolution of better fruit fly individuals. Zheng et al. [36] presented a novel fruit fly optimization algorithm to solve the semiconductor final testing scheduling problem. A collaborative search process was designed, including improved priority operation crossover and improved multi-point protection crossover, on the basis of two famous flexible job shop scheduling structures. The local search ability of FOA is reliable, but it is easy to cause a loss of population diversity, leading to premature convergence of the algorithm. Especially for problems with a large search space and many locally optimal solutions, the fruit fly optimization algorithm struggles to obtain a satisfactory solution.

The main contributions of this article are reflected in the following aspects:

- The basic problem definition of multifunctional heterogeneous UAV collaborative mission planning is given. The Dubins airplane model is introduced in the mission planning framework to generate the three-dimensional flight trajectory of each UAV.
- In order to improve the global search ability of the fruit fly optimization algorithm, multiple swarm mechanisms are introduced. In addition, two smell-based search strategies are designed to prevent the algorithm from prematurely falling into a local optimum. A two-strategy switching method is designed, and each fruit fly swarm is adaptively assigned the next iteration’s smell-based search strategy according to the results of each iteration.
- The application of the fruit fly optimization algorithm in the field of UAV mission planning expands its application field.

The remainder of this paper is organized as follows: Section 2 formulates the MFHCMPP using a mixed integer linear programming model, and the Dubins airplane model is introduced. The basic FOA is introduced in Section 3, and then the MFOA for solving the MFHCMPP is proposed in Section 4. The results of numerical tests and comparisons are provided in Section 5. Finally, the conclusions of this paper and future work are given in Section 6.

## 2. Problem Formulation

### 2.1. Task and UAV

Suppose that a mission contains a series of diverse tasks to be performed by the UAVs, such as photographing a building, data gathering from sensor network, or monitoring fixed positions. The mission is defined as a T-sized set $T=\left\{T_{1},T_{2},\ldots ,T_{T}\right\}$, where each task $T_{t}\left(t=1,2,\ldots ,T\right)$ involved in the mission scenario is asked to be performed only once. In this paper, the duration of each task performed by the UAV is ignored, and the task is deemed completed when the UAV arrives at the task location. In addition, a precedence constraint exists between some tasks, which is set by the human operator. Specifically, some tasks cannot be executed unless the predecessor tasks are performed. Let $T_{t}^{\prime }$ be the predecessor task of the task $T_{t}$, such that the execution time of $T_{t}$ should be later than the execution time of $T_{t}^{\prime }$, that is, $d_{T_{t}}>d_{T_{t}^{\prime }}$. Let $L=\left\{L_{1},L_{2},\ldots ,L_{T}\right\}$ be a set of T task positions, where $L_{t}=\left(x_{t},y_{t},z_{t}\right)\in {\mathbb{R}}^{3}$ stands for the task position of task $T_{t}$. The termination condition is defined as that the task position is visited by the UAV performing the task.

Let $U=\left\{U_{1},U_{2},\ldots ,U_{W}\right\}$ be the set of heterogeneous UAVs involved in the mission scenario. Each UAV has several functions with certain levels. The UAV heterogeneity is mainly reflected in the functions or functional level, related to the sensors or loads equipped on the UAVs. For example, UAV $U_{1}$ is equipped with an infrared sensor and a high-resolution vision sensor, and UAV $U_{2}$ is equipped with synthetic aperture radars and a low-resolution vision sensor. It is obvious that the UAV $U_{1}$ has the functions of detecting infrared information, target recognition, and photographing, but it does not have all-weather remote sensing functions. UAV $U_{2}$ has all-weather remote sensing and target recognition functions, but the visual sensor’s resolution limits the target recognition functional level of UAV. It may not satisfy the requirement of specific tasks, such as the recognition of small-size targets or complex background targets. In summary, the premise for a task to be executed is that the UAV meets the task’s function and corresponding level requirements.

To make it clearer, suppose that W heterogeneous UAVs have N functions and each function is divided into K levels. Let $U_{w}\Rightarrow F_{n}^{k}$ represent the function of UAV $U_{w}\in U$ and the corresponding functional level, 1≤n≤N, 1≤k≤K, 1≤w≤W. Let $T_{t}\Rightarrow SF_{n}^{k}$ denote the function and corresponding functional level of the UAV required to perform each task $T_{t}\in T$. In other words, the task can be performed unless the UAV has the function n, and the functional level is no less than k, where n is the type of function and k is the corresponding functional level.

**Figure 1:**
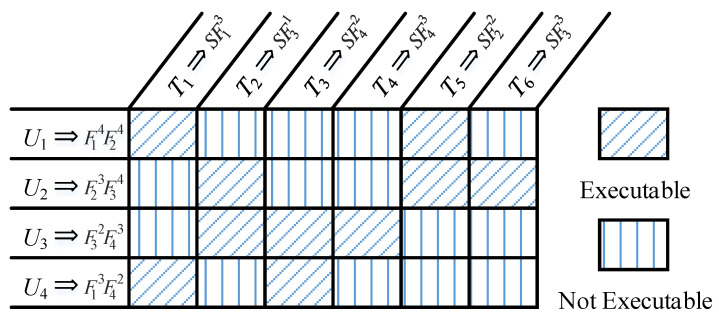
Examples of functional constraints.

Examples of functional constraints are presented in Figure 1. Taking $T_{4}$ as an example, it requires that the UAV performing the task has function 4, and the corresponding level of the function is not less than 3. Thus, $U_{1}$ and $U_{2}$ cannot be assigned to perform $T_{4}$, since $U_{1}$ and $U_{2}$ have no function 4. In addition, $U_{4}$ has function 4, but it also cannot perform $T_{4}$, because the function level corresponding to function 4 is less than 3.

### 2.2. UAV Trajectory Generation Model

In this paper, the Dubins airplane model [26] is introduced to generate the optimal flight path satisfying the kinematic constraints of the UAV in three-dimensional space. The spatial state of UAVs can be expressed by the following configuration:

(1) q=(p,θ,ψ),

where p=(x,y,z) is the position of the UAV in three-dimensional space, $p\in {\mathbb{R}}^{3}$, ψ is the heading angle of the UAV, and θ is the flight-path angle. The UAV’s kinematic equations are expressed as follows:

(2) $$\{\begin{array}{l} \dot{x}=v_{u}\cdot \cos\psi \cdot \cos\theta \\ \dot{y}=v_{u}\cdot \sin\psi \cdot \cos\theta \\ \dot{z}=v_{u}\cdot \sin\theta \\ \dot{\psi }=c_{\psi }\cdot v_{u}\cdot \rho_{\min}^{-1} \end{array},$$

where $v_{u}$ is the constant flight speed of the UAV, $\rho_{\min}$ is the minimum turning radius for the UAV, and $c_{\psi }$ is the steering control command such that $c_{\psi }\in \left[-1,1\right]$.

The Dubins airplane model was introduced as a trajectory planning model, as an extension of the Dubins car model. The Dubins car model is used for minimum-length trajectory planning in the two-dimensional plane from the start configuration $\left(x_{start},y_{start},\psi_{start}\right)$ to the final configuration $\left(x_{final},y_{final},\psi_{final}\right)$. The minimum-length trajectory generated by the Dubins car model is represented by six forms, such that the Dubins car set is defined by

(3) D={LSL,LSR,LRL,RSL,RSR,RLR},

where L is an anticlockwise arc of a circle with radius $\rho_{\min}$, R is a clockwise arc of a circle with radius $\rho_{\min}$, and S is a straight line segment. Taking RSL and RLR as examples, RSL represents a trajectory composed by a clockwise arc followed by a straight line segment followed by an anticlockwise arc, while RLR represents a trajectory composed by a clockwise arc followed by an anticlockwise arc followed by a clockwise arc.

Based on the Dubins car model, the Dubins airplane model extends the motion to the vertical plane to generate a minimum-length trajectory in three-dimensional space. The Dubins airplane model divides the space into three cases: low altitude, medium altitude, and high altitude. For low altitude, the trajectory can be obtained by adjusting the flight angle of the UAV on the basis of the trajectory generated by the Dubins car model. For high altitude, a certain number of additional helix segments need to be added to the start or end arcs to give the UAV enough distance to adjust the altitude. For the minimum altitude case, the minimum-length trajectory can be obtained without adding several helix segments, as an incomplete helix segment is enough. More specific implementation details be found in references [26,28]. The minimum length trajectory forms generated by the Dubins airplane model are given as follows:

(4) $$D^{+}=\left\{RLSR,RLSL,LRSR,LRSL,RSLR,RSRL,LSLR,LSRL\right\}$$

It is worth noting that, in order to achieve the minimum-length trajectory, the optimal heading angle $\psi^{*}$ and optimal flight-path angle $\theta^{*}$ of the UAV flying between various targets need to be solved. The optimal flight-path angle $\theta^{*}$ can be obtained by solving the Dubins airplane model. As the input of the Dubins airplane model, the optimal heading angle cannot be optimized directly by the model. We use the method of uniform sampling to discretize the heading angle for approximately solving the optimal heading angle, such that the heading angle sample set is denoted as

(5) $$H=\left\{\psi_{i}:\psi_{i}=2\pi i/N_{\psi },i=0,1,\ldots ,N_{\psi -1}\right\},$$

where $N_{\psi }$ is a positive integer, representing the number of samples. The approximate optimal heading angle is selected from the set of heading angle samples.

### 2.3. Mixed Integer Linear Programming Model

In this paper, we formulate the MFHCMPP using a mixed integer linear programming model. The objective is to optimize the cumulative flight distance of all UAVs for mission execution and the makespan. The mathematical model is as follows:

(6) $$J=\alpha \cdot \sum_{t=1}^{T}\sum_{i=1}^{W}\sum_{j=1}^{N_{\psi }}X_{i,T_{t}}^{\psi_{j}}\cdot L_{i,T_{t}}^{\psi_{j}}/v_{u}^{i}+\beta \cdot \max\sum_{t=1}^{T}\sum_{j=1}^{N_{\psi }}X_{i,T_{t}}^{\psi_{j}}\cdot L_{i,T_{t}}^{\psi_{j}}/v_{u}^{i},$$

(7) $$f_{T_{t}^{\prime }}\leq f_{T_{t}};\forall t\in T,$$

(8) $$y_{T_{t},i}=1;t\in T,\exists i\in U,$$

(9) $$\sum_{i=1}^{W}X_{i,T_{t}}^{\psi_{j}}\cdot F_{n}^{k}\geq Y_{T_{t},n,k}\cdot SF_{n}^{k};\forall t\in T,$$

(10) $$\sum_{t=1}^{T}y_{T_{t},i,d}\leq 1;\forall i\in U,\exists t\in T,d.$$

The cost function is shown in Equation (6). The objective is to minimize the makespan and the total mission time at the same time. In Equation (6), α and β are positive real values and their sum is 1; they are used to weigh the importance of the makespan and total mission time. In this paper, α is set to 0.6 and β is set to 0.4. $X_{i,T_{t}}^{\psi_{j}}$ is a binary decision variable. If $U_{i}$ performs $T_{t}$ with heading angle $\psi_{j}$, $X_{i,T_{t}}^{\psi_{j}}=1$; otherwise, $X_{i,T_{t}}^{\psi_{j}}=0$. $L_{i,T_{t}}^{\psi_{j}}$ represents the trajectory length of the UAV *i* approaching the location of task $T_{t}$ with the heading angle $\psi_{j}$. $v_{u}^{i}$ is the flight speed of $U_{i}$. The constraint in Equation (7) guarantees the precedence constraints of the task, where $f_{T_{t}^{\prime }}$ is the performing time of $T_{t}^{\prime }$ and $f_{T_{t}^{}}$ is the performing time of $T_{t}$. The constraint in Equation (8) ensures that each task can be handled only once, $y_{T_{t},i}$ is a binary decision variable. If $T_{t}$ is performed by $U_{i}$, $y_{T_{t},i}=1$; otherwise, $y_{T_{t},i}=0$. The constraint in Equation (9) guarantees that the UAV handling the task needs to meet the standard functional requirements of the task, where $Y_{t,n,k}$ represents that $T_{t}$ can be executed by the UAV, with function n and a corresponding functional level no less than k. The constraint in Equation (10) guarantees that each UAV can handle at most one task at a time, where $y_{T_{t},u,d}$ is a binary decision variable. If $T_{t}$ is being performed by $U_{i}$ at time d, $y_{T_{t},u,d}=1$; otherwise, $y_{T_{t},u,d}=0$.

## 3. Fruit Fly Optimization Algorithm

The fruit fly optimization algorithm is an emerging swarm intelligent optimization algorithm, which is based on the bionics principle of fruit fly foraging behavior. The excellent foraging behavior of a fruit fly mainly depends on its keen sense of smell and vision. The fruit fly can smell food sources far away and estimate the approximate location. The fruit fly can judge the odor concentration in the air and fly in the best possible direction of the food source. After approaching the food source, the fruit fly can discover other fruit flies’ locations or find the food source directly using vision.

The fruit fly optimization algorithm uses the principles of olfaction and visual search in the predation process of fruit flies to design a smell-based and vision-based search operation. The search for the solution is achieved mainly by alternating the smell-based and vision-based search operation. The process of the original fruit fly optimization algorithm is as follows:

- Step 1: Initialization. The swarm center of the fruit flies, the size of the fruit fly swarm, and the maximum number of iterations are initialized.
- Step 2: Smell-based search operation.
- Step 2.1: Several fruit fly individuals are randomly generated near the center of the fruit fly swarm. The number of individuals is the size of the fruit fly swarm.
- Step 2.2: The individuals of the fruit fly swarm are evaluated, the corresponding odor concentration value is calculated, and the odor concentrations are ranked.
- Step 3: Vision-based search operation.
- Step 3.1: The individual with the best odor concentration is selected.
- Step 3.2: The center of the fruit fly swarm is updated.
- Step 4: If the termination condition is reached, the current optimal solution is taken as the final solution. Otherwise, the process returns to step 2.

## 4. Multi-Swarm Fruit Fly Optimization Algorithm

### 4.1. Procedure of MFOA

The local search ability of FOA is outstanding, but its global search ability is relatively weak. Thus, it is easy to fall into a local optimum, leading to premature convergence. In the process of iterative optimization, the swarm of FOA updates the center according to the location of the optimal individual of the fruit flies. Once the swarm center’s location is determined, all the fruit flies in the swarm will fly toward the center of the swarm. This process leads to diversity loss, resulting in the FOA falling into a local optimal solution.

Two improved mechanisms are introduced to maintain the diversity and improve the global search ability of the algorithm: the multi-swarm mechanism and the dual search strategy mechanism. In the MFOA, the multi-swarm mechanism is used to determine multiple swarm centers, which effectively avoids the individual fruit flies gathering in one center, protecting the algorithm from falling into a local optimum. The dual search strategy mechanism includes a large-scale search strategy and a local search strategy, applied in the smell-based stage. The fruit flies have a larger fly radius with the large-scale search strategy, and the corresponding swarm focuses on the global search. For swarms with local search strategies, the fly radius of the fruit flies is small, focusing on the optimization of the current optimal solution. In each round of iterative optimization, the fruit fly swarms are divided into two groups according to a specific rule. Some of the swarms perform the large-scale search strategy, while the other swarms perform the local search strategy.

**Figure 2:**
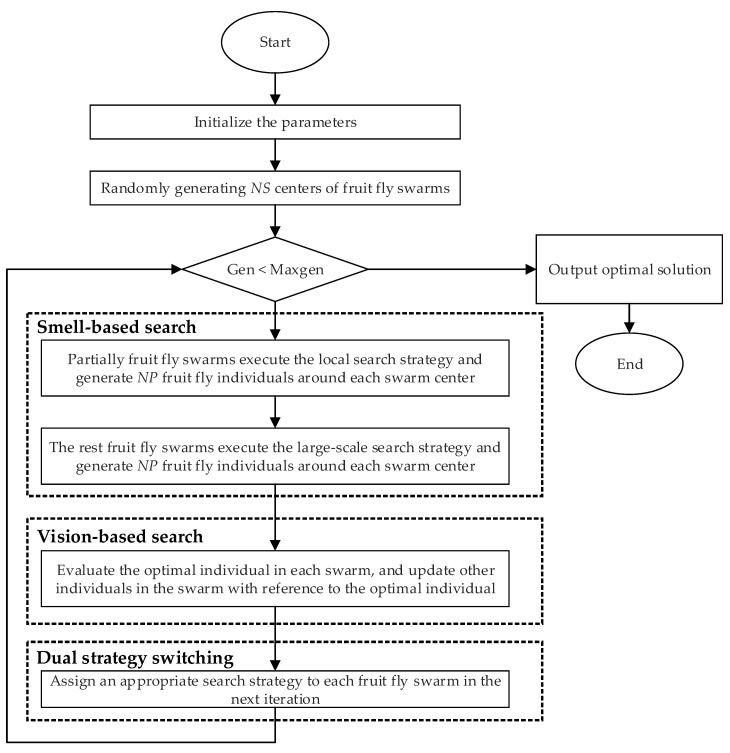
The flowchart of the multi-swarm fruit fly optimization algorithm (MFOA).

The flowchart of MFOA for solving the MFHCMPP is shown in Figure 2. It can be seen that the MFOA mainly consists of four phases, i.e., initialization, smell-based search, vision-based search, and dual search strategy switching. Firstly, the number of fruit fly swarms *NS*, the size of fruit fly swarm *NP*, and the maximum number of iterations *Maxgen* are initialized. Then, based on the number of fruit fly swarms, *NS* fruit fly individuals are generated as the centers of the swarms. Each individual fruit fly represents a feasible solution to this problem. In the smell-based phase, some fruit fly swarms execute the local search strategy, while the remaining fruit fly swarms execute the large-scale search strategy. It is worth noting that all fruit fly swarms are set to perform the local search strategy in the first iteration. The search strategy of each fruit fly swarm in the subsequent iterations is determined by the dual strategy switching process. For the vision-based search stage, the new centers of the swarms are generated using the greedy selection strategy. In other words, during each iteration, the fruit fly individual with the optimal odor concentration in a swarm is designated as the new center of the swarm. Finally, the appropriate search strategy is assigned to the next iteration of the swarm. The iterative optimization process is repeated until the termination condition is reached.

### 4.2. Encoding

**Figure 3:**
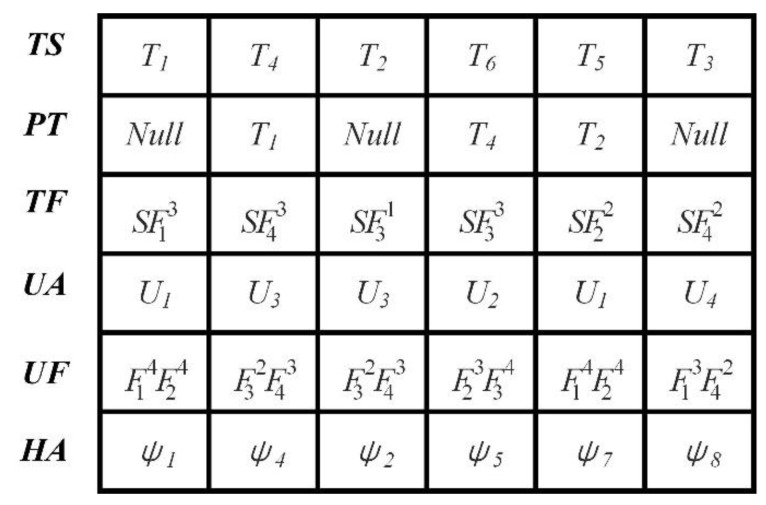
Encoding example of a fruit fly individual.

Each fruit fly individual represents a feasible solution for the MFHCMPP. Thus, the fruit fly individual encoding is the precondition to solve the problem. The fruit fly individual encoding is designed to be composed of six lists, i.e., task sequencing list (TS), predecessor task list (PT), task function requirements list (TF), heterogeneous UAV allocation list (UA), heterogeneous UAV function list (UF), and heading angle list (HA). The encoding example of a fruit fly individual is shown in Figure 3. The TS list represents the task execution order. The PT list indicates the predecessor task of the corresponding tasks in the TS list (e.g., in Figure 3, the predecessor task of task $T_{4}$ is task $T_{1}$, while task $T_{1}$ has no predecessor tasks, which is represented by *Null* in the PT list). The TF list represents the functional constraint of the task (e.g., in Figure 3, task 5 cannot be performed unless the UAV has function 3 and the function level is not less than the standard level 2). The UA list denotes the heterogeneous UAV assignment for each task. The HA determines the terminal heading angle when the UAV approaches the task position. The mathematical description of the lists is given in Equations (11)–(16).

(11) TS=[ts(1),ts(2),…,ts(T)],

(12) PT=[pt(1),pt(2),…,pt(T)],

(13) TF=[tf(1),tf(2),…,tf(T)],

(14) UA=[ua(1),ua(2),…,ua(T)],

(15) UF=[uf(1),uf(2),…,uf(T)],

(16) HA=[ha(1),ha(2),…,ha(T)].

To be specific, *ts*(*i*)(*i* = 1,2,…,*T*) represents the *i*-th task to be assigned, *pt*(*i*)(*i* = 1,2,…,*T*) denotes the predecessor task of *ts*(*i*), *tf*(*i*)(*i* = 1,2,…,*T*) indicates the standard functional requirements of the *i*-th task, *ua*(*i*)(*i* = 1,2,…,*T*) implies the UAV assigned to the *i*-th task, *uf*(*i*)(*i* = 1,2,…,*T*) represents the functions of *ua*(*i*), and *ha*(*i*)(*i* = 1,2,…,*T*) indicates the terminal heading angle when the *ua*(*i*) approaches the *i*-th task’s location.

### 4.3. Initialization

The number of fruit fly swarms is *NS*, and the number of fruit flies in each swarm is *NP.* Thus, *NS* × *NP* fruit fly individuals should be generated. Each fruit fly individual is randomly generated.

Specifically, the TS list is generated by randomly selecting a task from the set of tasks T and inserted randomly into the TS list without violating the task priority constraints. The selection and insertion process is repeated until all tasks are inserted into the TS list. The PT list and the TF list are generated according to the task priority constraints and the task functional constraint, respectively. The UA list is generated by randomly allocating a UAV for each task, with the premise that the function of the UAV has to meet the task functional constraints. The UF list is generated according to the function of UAV and the corresponding function level. The HA list is generated by randomly selecting an approach heading angle from the heading angle sample set H for the UAV.

### 4.4. Smell-Based Search

The smell-based search stage, as the core of FOA, greatly affects the efficiency of problem solving and the quality of the solution. In the smell-based search stage of this paper, we drive the fruit fly swarm through the dual search strategy mechanism, i.e., incorporating a large-scale search strategy and a local search strategy. The purpose is to maintain the diversity of the swarm while ensuring the convergence rate of the problem.

#### 4.4.1. Local Search Strategy

**Figure 4:**
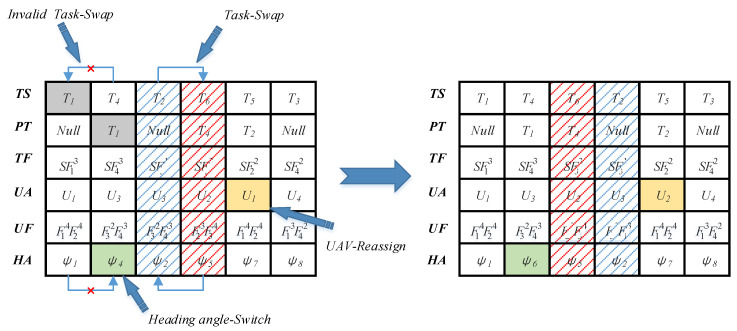
Example of the local search strategy.

The local search strategy focuses on further optimizing the advantage of fruit fly individuals. The operation operators do not change the position of individual fruit flies significantly, because this would cause the fruit flies to be too far away from the current position and reduce the search efficiency. An example of the local search strategy is shown in Figure 4. The specific operators are given as follows:

- Task Exchange: The task exchange operation adopts the neighborhood exchange method to exchange the columns of two adjacent tasks without priority constraints (e.g., in Figure 3, the element of the PT list corresponding to the task $T_{2}$ is “*Null*”, which means that $T_{2}$ has no predecessor task. The element of the PT list corresponding to the task $T_{6}$ is “$T_{4}$”; thus, $T_{4}$ is the predecessor task of the task $T_{6}$. Task $T_{2}$ and task $T_{6}$ have no priority constraints; thus, the task exchange operator can be used for operation. The columns of tasks $T_{1}$ and $T_{4}$ cannot be exchanged because the predecessor task of $T_{4}$ is task $T_{1}$).
- UAV Reassign: The UAV reassign operation randomly selects a task and reassigns it to a new UAV that meets the task function constraints (e.g., in Figure 3, task $T_{5}$ requires that the UAV performing the task has the function 5 and the standard level is not less than 2. Therefore, the UAV reassign operator traverses the UF list and select another UAV that meets the task function constraints for reassignment).
- Heading Angle Switch: The heading angle switch operation randomly selects an approach heading angle for resampling according to $N_{\psi }$.

#### 4.4.2. Large-Scale Search Strategy

**Figure 5:**
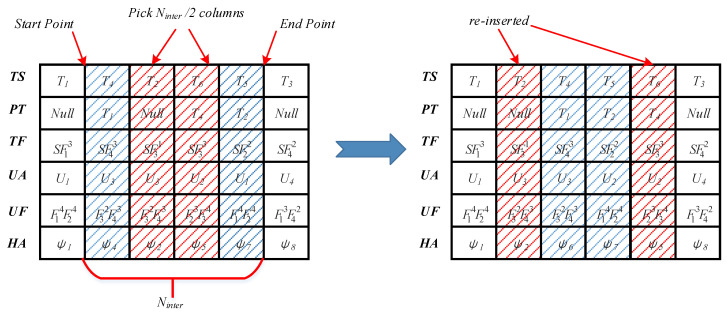
Example of large-scale search strategy.

The large-scale search strategy focuses on keeping the fruit fly individuals with poor performance away from the current position. An example of the large-scale search strategy is shown in Figure 5. The specific operators are given as follows:

- Interval task reordering: The interval task reordering operator randomly selects the starting point and the ending point in the TS list. The number of tasks included between the start point and the end point is $N_{\mathrm{int}er}$, where $N_{\mathrm{int}er}$ is even and $4\leq N_{\mathrm{int}er}\leq T-4$. Then, $N_{\mathrm{int}er}/2$ columns are taken out from the fruit fly individuals and reinserted randomly without violating the task priority constraints.
- Multi-UAV Reassign: The multi-UAV reassign operation randomly selects $N_{reassign}$ tasks and reassigns them to new UAVs that meet the task function constraints.
- Multi-Heading Angle Switch: The heading angle switch operation randomly selects $N_{\mathrm{switch}}$ approach heading angles for resampling according to $N_{\psi }$.

### 4.5. Vision-Based Search

In the vision-based search stage, each fruit fly swarm evaluates the fruit fly individuals to obtain each fruit fly individual’s odor concentration value. In order to update the central position of each fruit fly swarm, a greedy selection strategy is adopted. The individual’s position with the optimal odor concentration in each fruit fly swarm is updated to the new center of the fruit fly swarm. It is worth noting that, if the fruit fly individual’s position with the optimal odor concentration is the same as the center of the current fruit fly swarm, the position of the center of the fruit fly swarm is not updated.

**Figure 6:**
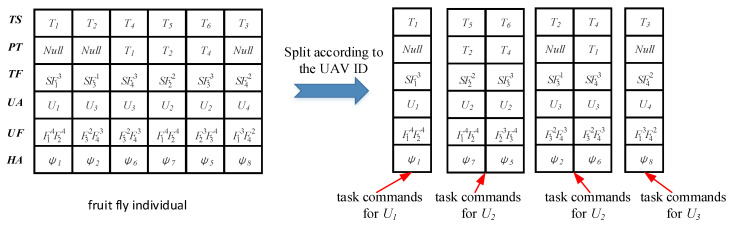
Process of UAV task command generation.

The odor concentration value of each fruit fly individual is determined by the makespan and the total mission time. Thus, to calculate the odor concentration value, the task commands for each UAV should be generated. The process of UAV task command generation is illustrated in Figure 6. The fruit fly individual is split according to the UAV identifier (ID). Taking $U_{2}$ as an example, its task command is to execute $T_{5}$ and $T_{6}$ in sequence. The heading angle of $U_{2}$ is designated as $\psi_{7}$ when it approaches the location of $T_{5}$. Then, $U_{2}$ leaves the location of $T_{5}$ with the heading angle $\psi_{7}$ and approaches the location of $T_{6}$ with the heading angle $\psi_{5}$. According to the task commands of each UAV and the Dubins airplane model, the flight time of each UAV can be calculated. Furthermore, the odor concentration value of the fruit fly individual, i.e., the makespan and the total mission time, can be determined.

Next, taking a swarm of multiple fruit fly swarms as an example to illustrate the greedy selection strategy, once the odor concentration values of all individuals in the fruit fly swarm are calculated, the greedy selection strategy compares the odor concentration values of all fruit flies and selects the fruit fly with the best odor concentration value as the new swarm center. In the next iteration of the process, the center of the fruit fly swarm executes a smell-based search NP times to generate *NP* new fruit fly individuals. A more detailed explanation of the vision-based search can be found in Reference [37].

### 4.6. Dual Strategy Switching

The dual strategy switching process is an important process that controls the smell-based search strategy of all fruit fly swarms. The local search strategy and the large-scale search strategy are assigned to *NS* fruit fly swarms. The process of the dual strategy switching method assigns the search strategy to each fruit fly swarm as follows:

- Step 1: The odor concentration value of each fruit fly center $F_{i}$ is calculated, where 1≤i≤NS.
- Step 2: The average odor concentration value of all the fruit fly swarm centers $F_{avg}$ is calculated, where i=1.
- Step 3: The odor concentration value $F_{i}$ is compared with the average odor concentration value $F_{avg}$ of all fruit fly swarm centers. If $F_{i}$ is larger than the average odor concentration value $F_{avg}$, then the local search strategy is assigned to the fruit fly swarm; otherwise, the large-scale search strategy is assigned.
- Step 4: Let i=i+1. If i=NS+1, it means that the search strategy of all fruit fly swarms is assigned, and the dual strategy switching process is completed. Otherwise, to the process returns to step 3.

The average odor concentration value of all the fruit fly swarm centers can be calculated as follows:

(17) $$F_{avg}=\sum_{i=1}^{NS}F_{i}/NS,$$

where $F_{avg}$ is the average odor concentration of the center of all fruit fly swarms, and $F_{i}$ represents the center odor concentration of the fruit fly swarm i (1≤i≤NS).

## 5. Results

### 5.1. Sample Run

In order to test the feasibility of the solution, a simple example experiment scene is given. The example scene is generated in three-dimensional (3D) space and contains three multi-functional heterogeneous UAVs and nine tasks. As shown in Table 1, the UAVs in the scene differ in terms of function type, function level, and mobility.

Table 2 shows the task settings of task 1 to task 4, and Table 3 shows the task settings of task 5 to task 9. The task settings are composed of four parts: task location, task height, predecessors, and functional requirements. Taking task 3 as an example, its space configuration is composed of task location and task height, and the UAV can access it at any flight path angle and heading angle. Its predecessor is task 1, which means that the UAV must access it after task 1 is completed.

**Figure 7:**
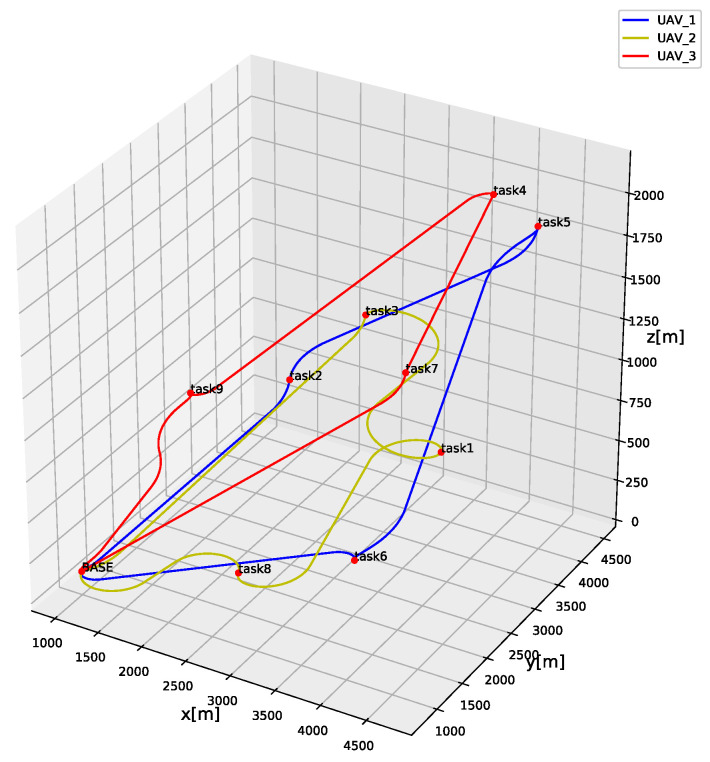
The three-dimensional trajectories of multi-UAVs.

The MFOA parameters are set to *NS* = 30, *NP* = 20, and $N_{\psi }$ = 100. In order to verify the generation of three-dimensional trajectories, the three-dimensional trajectories of multifunctional heterogeneous UAV performing tasks are shown in Figure 7. The blue line represents the three-dimensional trajectory of UAV 1’s mission. After starting from the base, it performs task 9, task 4, and task 7, before returning to the base. The yellow line represents the three-dimensional trajectory of UAV 2’s mission. After starting from the base, it performs task 3, task 1, and task 8, before returning to the base. The red line represents the three-dimensional trajectory of UAV 3’s mission. After it departs from the base, it executes task 9, task 4, and task 7, before returning to the base. The cumulative mission completion time for all UAVs is 772.26 s. The mission completion times for UAV 1, UAV 2, and UAV 3 are 260.75 s, 284.65 s, and 226.86 s, respectively. It can be seen from Figure 7 that all tasks are assigned to multifunctional UAVs that meet the task functional requirements.

Regarding the timing of tasks, the predecessor of task 1 is task 3, and both tasks are executed by UAV 2. The timing constraints are satisfied. As shown in Table 3, the predecessor of task 5 in the initial task setting is task 9. Task 9 is executed by UAV 3 at 66.55 s, and task 5 is executed by UAV 1 at 139.28 s. Therefore, the timing constraints between task 5 and task 9 are also satisfied.

The MFOA algorithm was verified in this section for 3D trajectory generation problems, timing constraint processing, and function satisfaction allocation. Next, the performance of the MFOA algorithm is evaluated.

### 5.2. MFOA Performance Analysis

#### 5.2.1. Experimental Set-Up

In order to verify the performance of the proposed MFOA algorithm, nine datasets with increasing complexity were designed. Table 4 shows the characteristics of the nine datasets, covering the number of tasks, the number of UAVs, the upper limit of function type, the upper limit of corresponding function level, the number of tasks with timing constraints, and the number of UAV functions. Taking dataset 1 as an example, it contained nine missions and three multifunctional heterogeneous UAVs. The number of functions of each multifunctional heterogeneous UAV was 2, the upper limit of the function type was 3, and the upper limit of the corresponding function level was 2. The functional requirements of the task were generated according to the functions of the UAV to ensure that all tasks could be executed.

On this basis, the datasets were divided into a small-scale task set, medium-scale task set, and large-scale task set. Among them, datasets 1–3 belonged to the small-scale task set, datasets 4–6 belonged to the medium-scale task set, and datasets 7–9 belonged to the large-scale task set. The coordinates of the task locations were randomly generated within the area of 8000 × 8000, and the height was randomly selected within the vertical range of [100, 5000]. The maximum bank angle and maximum flight-path angle were uniformly set to π/4 and π/6 degrees. The airspeed of the UAVs was uniformly set to 40–70 ms/s.

To test the performance of MFOA in different scale mission scenarios, the proposed MOFA algorithm was compared with the random search algorithm (RSM) and FOA algorithm in a specific small-scale mission scenario, medium-scale mission scenario, and large-scale mission scenario. The random search algorithm is usually used as a benchmark method for comparison to prove the effectiveness of new algorithms. It uses a random strategy to update the individuals in the population and selects the best individual as the starting point for the next iteration. In Reference [32], random search algorithm was used to compare the performance of algorithms. The scale of the RSM solution was consistent with FOA and MFOA. Initially, *NS* × *NP* candidate solutions were generated by random selection. In each iteration, RSM kept the candidate solution with the optimal objective value, and the remaining *NS* × *NP* − 1 candidate solutions were generated by the random selection strategy. For FOA, *NS* × *NP* initial solutions were randomly generated based on the random selection strategy. All fruit fly individuals were updated based on the only fruit fly center. The smell-based search stage for FOA adopted the local search strategy like the MFOA, and the visual search stage adopted the greedy selection strategy.

#### 5.2.2. Small-Scale Mission Scenario Test

As shown in Table 4, dataset 1 was selected as the test scenario to verify the performance of the MFOA algorithm in the small-scale mission scenario. The operating parameters of the RSM, FOA, and MFOA algorithms are shown in Table 5.

**Figure 8:**
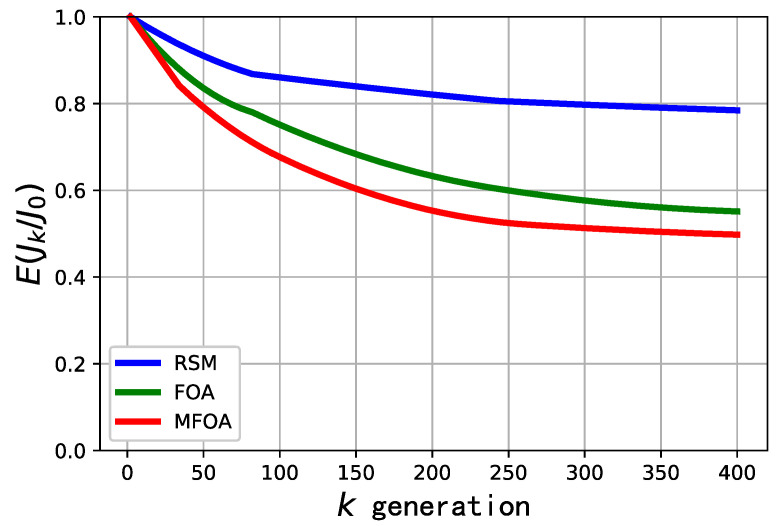
Comparison of convergence efficiency of RSM, FOA, and MFOA in the small-scale mission scenario (dataset 1).

The comparison of convergence efficiency for the RSM, FOA, and MFOA in the small-scale mission scenario is shown in Figure 8. The convergence efficiency of the algorithms is described by $E\left(J_{k}/J_{0}\right)$, where $J_{0}$ represents the optimal odor concentration in the initial population, and $J_{k}$ represents the optimal odor concentration in the population of the *k*-th generation. As shown in Figure 8, MFOA had the fastest convergence rate and RSM had the slowest convergence rate in the small-scale mission scenario. The convergence performance of MFOA was slightly better than that of FOA.

**Figure 9:**
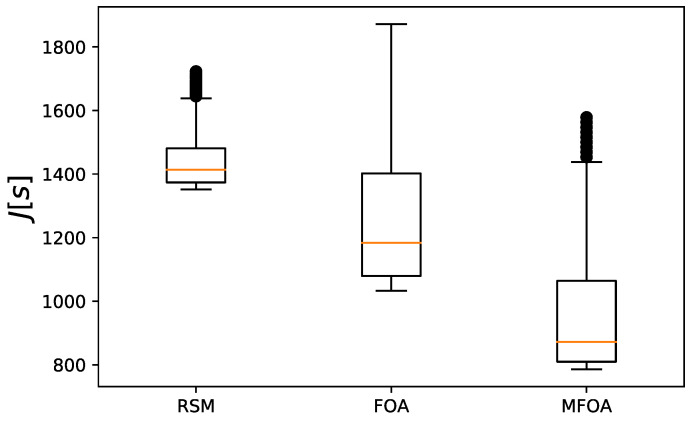
Comparison of objective value distribution of RSM, FOA, and MFOA in the small-scale mission scenario (dataset 1).

The objective value distribution of the RMS, FOA, and MFOA for the small-scale mission scenario is shown in Figure 9. The RSM algorithm had the highest concentration of target values and the lowest edge value, indicating that the RSM algorithm had low optimization efficiency and fell prematurely into a local optimum. The FOA algorithm had a faster convergence speed and stronger optimization performance when processing the small-scale mission scenario, but the ability to jump out of the local optimal value was weaker. Compared with the other two algorithms, the objective value of MFOA was concentrated in a lower value, indicating that the optimization effect was better when processing the small-scale task scenario than the other two algorithms.

#### 5.2.3. Medium-Scale Mission Scenario Test

As shown in Table 4, dataset 4 was selected as the test scenario to verify the performance of the MFOA algorithm in the medium-scale mission scenario. The operating parameters of the RSM, FOA, and MFOA are shown in Table 6.

**Figure 10:**
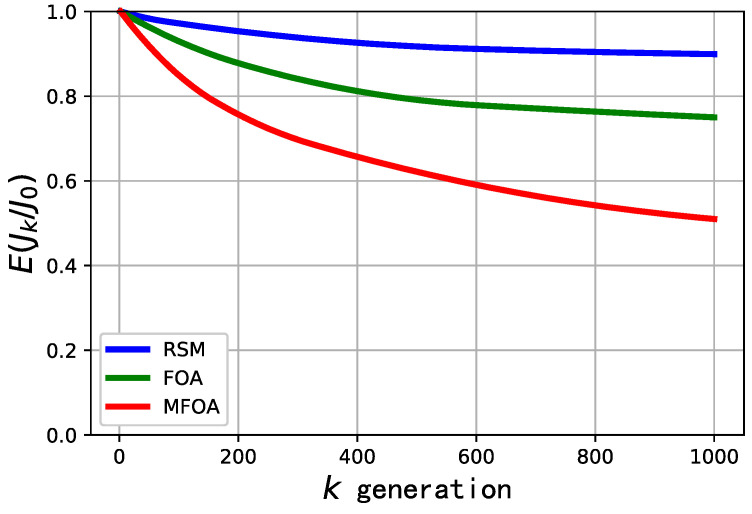
Comparison of convergence efficiency of RSM, FOA, and MFOA algorithms in the medium-scale mission scenario (dataset 4).

The comparison of convergence efficiency of the RSM, FOA, and MFOA in the medium-scale mission scenario is shown in Figure 10. The convergence performance of RSM was poor, and it was prematurely caught in a local optimum. In terms of optimizing the processing of large-scale task scenarios, the convergence performance of the FOA algorithm was weaker than when processing the small-scale mission scenario. The MFOA algorithm had better convergence performance when dealing with mid-scale problems, and, after 1000 iterations, the algorithm did not fully converge and still had a certain optimization space.

**Figure 11:**
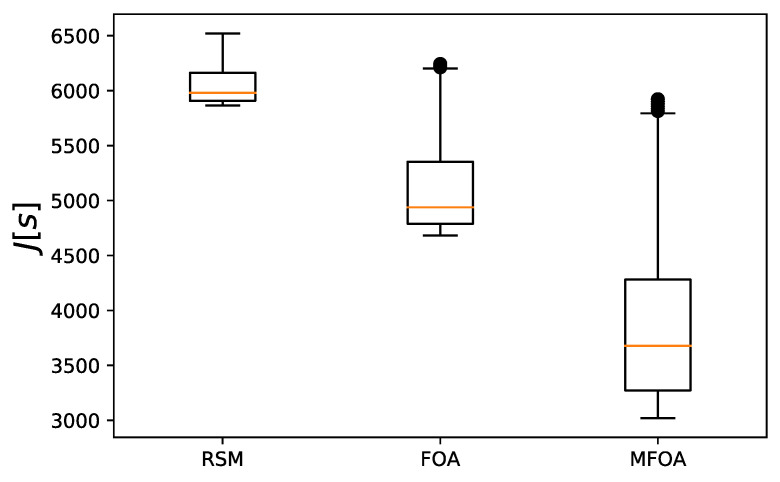
Comparison of objective value distribution of the RSM, FOA, and MFOA in the medium-scale mission scenario (dataset 4).

The objective value distribution of the RMS, FOA, and MFOA for the medium-scale mission scenario is shown in Figure 11. The lower edge value of RSM and FOA was higher, indicating that the optimization effect of the two algorithms when processing the medium-scale task scenario was weak. The median value of the target value of the MFOA algorithm was still far away from the lower edge value, indicating that the algorithm did not fall into a local optimum.

#### 5.2.4. Large-Scale Mission Scenario Test

As shown in Table 4, dataset 7 was selected as the test scenario to verify the performance of the MFOA algorithm in a large-scale mission scenario. The operating parameters of the RSM, FOA, and MFOA are shown in Table 7.

**Figure 12:**
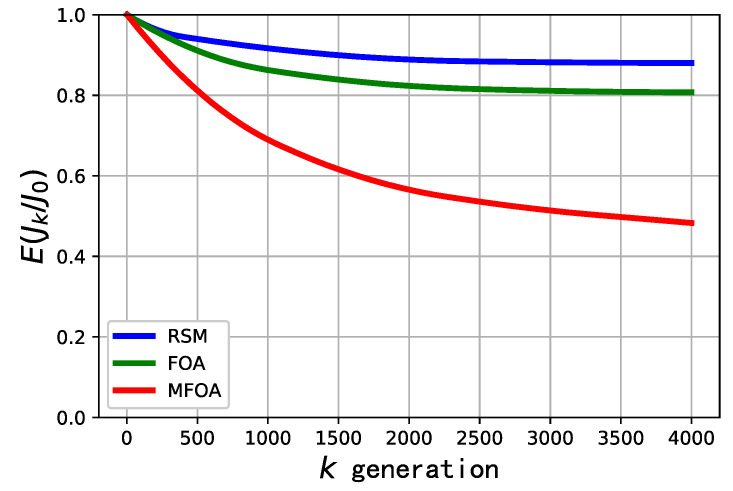
Comparison of convergence efficiency of the RSM, FOA, and MFOA in the large-scale mission scenario (dataset 7).

**Figure 13:**
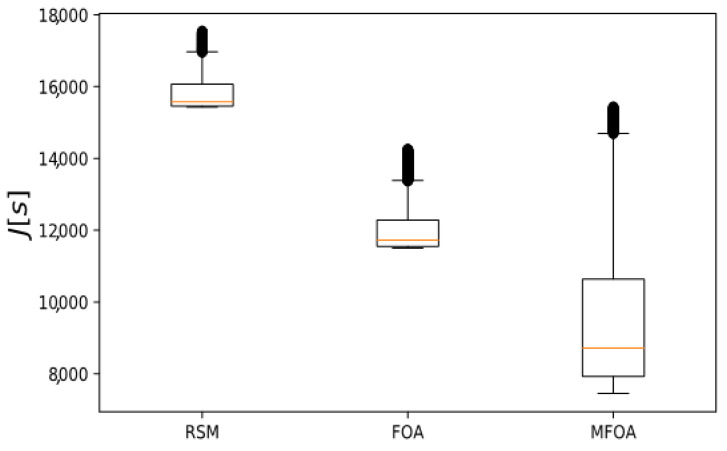
Comparison of objective value distribution of the RSM, FOA, and MFOA in the large-scale mission scenario (dataset 7).

The comparison of convergence efficiency of the RSM, FOA, and MFOA in the large-scale mission scenario is shown in Figure 12. The objective value distribution of the RMS, FOA, and MFOA for the large-scale mission scenario is shown in Figure 13. Due to the increased task complexity of the large-scale mission scenario, the convergence efficiency of RSM and FOA was low. When the algorithm converged, the lower edge of the objective value distribution was high, indicating that the optimization effect was poor. In the large-scale task scenario, the MFOA algorithm had obvious advantages over the other two algorithms, and the algorithm optimization performance was better. It shows that the MFOA algorithm is more suitable to deal with complex optimization problems with a large-scale search space.

### 5.3. Monte Carlo Stability Analysis

In order to test the stability of the MFOA algorithm, the stability of the MFOA algorithm was tested by Monte Carlo simulation and compared with RSM and FOA. The test dataset is shown in Table 4, which contains the small-scale mission scenario set, medium-scale mission scenario set, and large-scale mission scenario set. The FOA, RSM, and MFOA were run 1000 times for each mission scenario in the dataset shown in Table 4. In the Monte Carlo simulation, the algorithm parameter settings for the small-scale mission scenario, medium-scale mission scenario, and large-scale mission scenario are shown in Table 5, Table 6 and Table 7, respectively.

**Figure 14:**
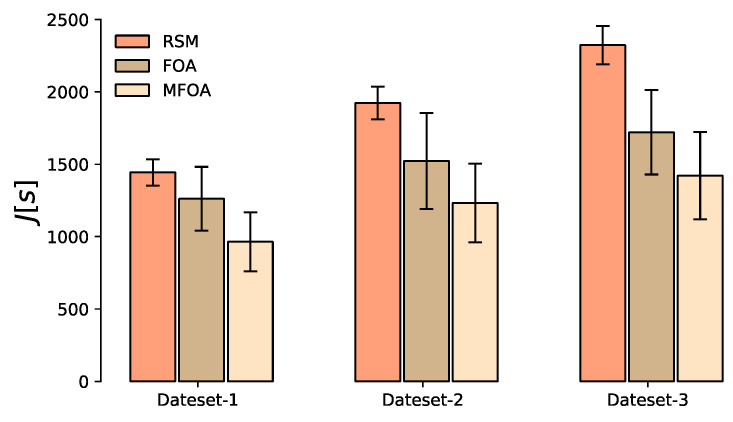
The results of the Monte Carlo simulation of RSM, FOA, and MFOA in the small-scale mission scenario (datasets 1–3).

**Figure 15:**
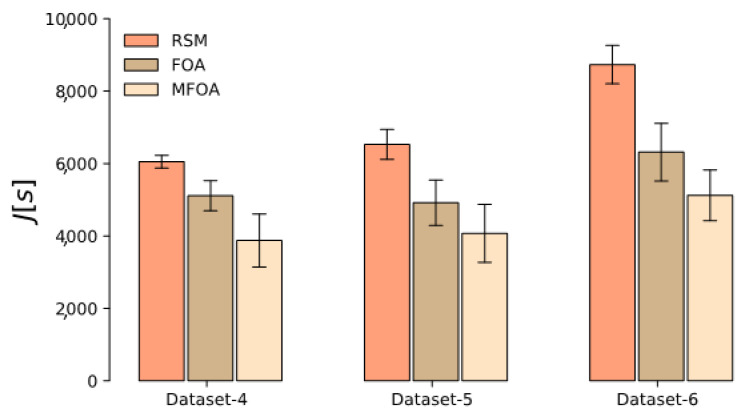
The results of the Monte Carlo simulation of RSM, FOA, and MFOA in the medium-scale mission scenario (datasets 4–6).

The results of the Monte Carlo simulation of RSM, FOA, and MFOA in the small-scale mission scenario represented by datasets 1–3 are shown in Figure 14. In Monte Carlo simulations based on datasets 1–3, the average objective value of MFOA was smaller than that of RSM and FOA, indicating that the MFOA is stable in small-scale mission scenarios. The results of the Monte Carlo simulation of RSM, FOA, and MFOA in the medium-scale mission scenario represented by datasets 4–6 are shown in Figure 15.

**Figure 16:**
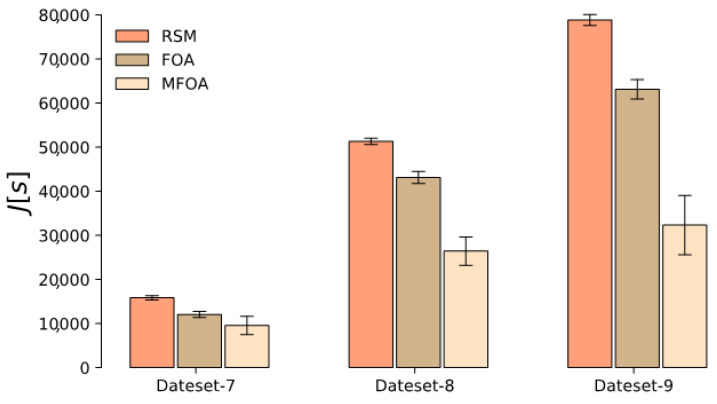
The results of the Monte Carlo simulation of RSM, FOA, and MFOA in the large-scale mission scenario (datasets 7–9).

Similar to the small-scale scenario, the optimization performance of MFOA in the medium-scale mission scenario was superior to RSM and FOA. The results of the Monte Carlo simulation of RSM, FOA, and MFOA in the large-scale mission scenario represented by datasets 7–9 are shown in Figure 16. From the analysis of Monte Carlo simulation results, it can be concluded that, due to the increase in the number of tasks, the number of drones, the heterogeneity of UAVs, and the timing correlations, the convergence efficiency of RSM and FOA was significantly reduced compared to the medium- and small-scale scenarios. Simulation results show that RSM and FOA are not suitable for large-scale mission scenarios. The optimization results of the MFOA algorithm in the large-scale task scenario still indicated good performance. It shows that the optimized performance of MFOA can be stable in small-, medium-, and large-scale mission scenarios.

## 6. Conclusions

In this paper, a multi-swarm fruit fly optimization algorithm with dual strategy switching (MFOA) was proposed to solve the multi-functional heterogeneous UAV cooperative mission planning problem with the criterion of simultaneously minimizing the makespan and the total mission time. The multi-functional heterogeneous UAV cooperative mission planning problem was considered to include three sub-problems of task assignment, task scheduling, and three-dimensional trajectory planning. Thus, a multi-list encoding architecture was proposed to represent the candidate solutions. The multi-swarm mechanism was introduced to enhance the global search performance of the basic fruit fly optimization algorithm. Meanwhile, in the smell-based search stage, a dual strategy collaborative search method was proposed to balance global and local search performance. In the vision-search stage, the decoding method of UAV task commands was given, and the greedy selection strategy was applied to update the center of the fruit fly swarm. A dual strategy adaptive switching method was given for adaptively assigning a multi-swarm search strategy in the smell-based search stage. A sample run was used to verify the effectiveness of the MFOA algorithm to deal with timing constraints, functional constraints, and the three-dimensional trajectory planning problem. Next, the performance of three algorithms (RSM, FOA, and MFOA) was compared in small-scale, medium-scale, and large-scale mission scenarios. the simulation results proved the superiority of MFOA in the three scenarios. Finally, Monte Carlo simulation experiments were carried out, and the experimental results showed that MFOA maintained its stability in small-, medium-, and large-scale mission scenarios.

Regarding further work in the future, we will consider the following aspects:

- More practical factors will be introduced in the mission planning problem, such as fuel constraints, UAV cost constraints, target value, and so on.
- Uncertain factors will be considered in the mission planning problem, such as the uncertainty of the target location or the uncertainty of the UAV’s flying speed.
- The fruit fly optimization algorithm will be further optimized to improve its performance.
- The complexity of the fruit fly optimization algorithm will be reduced, and new optimization mechanisms will be explored to test its performance in real-time mission planning problems.

## Figures and Tables

**Table 1:** Parameters of heterogeneous unmanned aerial vehicles (UAVs).

Parameters	No. UAV		
	$U_{1}$	$U_{2}$	$U_{3}$
Base Location	[1000,1000]	[1000,1000]	[1000,1000]
Base Height	100	100	100
Function	$\left[F_{2}^{3},F_{3}^{2}\right]$	$\left[F_{1}^{2},F_{3}^{3}\right]$	$\left[F_{1}^{3},F_{2}^{2}\right]$
Airspeed (m/s)	50	40	60
Maximum Bank Angle	π/4	π/4	π/4
Maximum Flight-Path angle	π/6	π/6	π/6

**Table 2:** Parameters of tasks 1–4.

Parameters	No. Task			
	$T_{1}$	$T_{2}$	$T_{3}$	$T_{4}$
Task Location	[3700,3400]	[2500,2500]	[2500,4000]	[3700,4400]
Task Height	600	1100	1100	1900
Predecessor Task	$T_{3}$	Null	Null	Null
Function Requirement	$F_{1}^{2}$	$F_{2}^{2}$	$F_{1}^{1}$	$F_{1}^{2}$

**Table 3:** Parameters of tasks 5–9.

Parameters	No. Task				
	$T_{5}$	$T_{6}$	$T_{7}$	$T_{8}$	$T_{9}$
Location	[4700,3400]	[3800,1500]	[4500,1200]	[2500,1500]	[1800,1800]
Height	2100	500	1800	200	1100
Predecessor Task	$T_{9}$	Null	Null	Null	Null
Function Requirement	$F_{3}^{2}$	$F_{2}^{2}$	$F_{1}^{2}$	$F_{3}^{1}$	$F_{2}^{1}$

**Table 4:** The characteristics of the datasets. ID—identifier.

Dataset ID	Tasks	UAVs	Number of UAV Functions	Function Types Upper Limit	Function Levels Upper Limit	Predecessor Tasks
1	9	3	2	3	2	3
2	12	3	2	3	3	5
3	16	4	2	4	3	4
4	25	6	3	4	3	7
5	28	6	3	5	4	9
6	35	8	3	5	4	12
7	50	12	4	6	5	13
8	72	14	4	6	5	15
9	85	16	4	6	6	15

**Table 5:** The operating parameters of the random search algorithm (RSM), FOA, and MFOA for the small-scale mission scenario test.

RSM			FOA			MFOA			
*NP*	$N_{\varphi }$	*Maxgen*	*NP*	$N_{\varphi }$	*Maxgen*	*NS*	*NP*	$N_{\varphi }$	*Maxgen*
300	100	400	300	100	400	50	6	100	400

**Table 6:** The operating parameters of the RSM, FOA, and MFOA for the medium-scale mission scenario test.

Dataset ID	RSM			FOA			MFOA			
	*NP*	$N_{\varphi }$	*Maxgen*	*NP*	$N_{\varphi }$	*Maxgen*	*NS*	*NP*	$N_{\varphi }$	*Maxgen*
1	800	100	400	800	100	400	80	10	100	400

**Table 7:** The operating parameters of the RSM, FOA, and MFOA for the large-scale mission scenario test.

Dataset ID	RSM			FOA			MFOA			
	*NP*	$N_{\varphi }$	*Maxgen*	*NP*	$N_{\varphi }$	*Maxgen*	*NS*	*NP*	$N_{\varphi }$	*Maxgen*
1	2000	100	400	2000	100	400	100	20	100	400
